# Construction and immunogenicity evaluation of a bivalent nanoparticle based on mi3 displaying porcine circovirus type 2 and type 3 capsid proteins

**DOI:** 10.3389/fvets.2026.1862938

**Published:** 2026-06-05

**Authors:** Baishi Lei, Jiale Du, Yiran Li, Yuchuan Yang, Peng Chen, Longhai Ji, Wuchao Zhang, Yunhang Zhang, Kuan Zhao, Wanzhe Yuan

**Affiliations:** 1 College of Veterinary Medicine, Hebei Agricultural University, Baoding, Hebei, China; 2 Hebei Key Laboratory of Analysis and Control of Zoonotic Pathogenic Microorganism, Baoding, Hebei, China; 3 National Research Center of Engineering and Technology for Veterinary Biologicals, Nanjing, Jiangsu, China

**Keywords:** mi3 nanoparticle, nanoparticle, porcine circovirus type 2, porcine circovirus type 3, SpyCatcher, SpyTag

## Abstract

Porcine circovirus type 2 (PCV2) and type 3 (PCV3) pose severe threats to swine health worldwide. PCV2 field strains continuously mutate with the co-circulation of multiple genotypes, resulting in potential immune escape from existing vaccines. PCV3, an emerging pathogen, currently has no commercially available vaccines. Clinically, PCV2-PCV3 co-infection is prevalent, thus highlighting an urgent need for a bivalent vaccine. This study aimed to construct a broad-spectrum, high-efficiency bivalent nanoparticle antigen (PCV2-PCV3 Nano) based on mi3 nanocages and to evaluate its immunological efficacy. First, amino acid sequences of PCV2 and PCV3 capsid (Cap) proteins were optimized through analysis of currently prevalent strains. SpyCatcher (SC) or SpyTag (ST) domains were genetically fused to mi3, PCV2 Cap, or PCV3 Cap, respectively. The fusion sequences were codon-optimized and heterologously expressed in *Escherichia coli*. After purification, PCV2 Cap and PCV3 Cap were covalently anchored onto mi3 nanocages via SC–ST bioconjugation, successfully constructing PCV2-PCV3 Nano. Immunization of BALB/c mice demonstrated that PCV2-PCV3 Nano significantly induced higher antibody levels and conferred immune protection against PCV2b, PCV2d, and PCV3 infections compared with conventional antigens and commercial PCV2 vaccines. This study establishes a critical foundation for the industrial application of PCV2-PCV3 Nano, and it provides technical support for the development of mi3-based multivalent vaccines.

## 1 Introduction

Porcine circovirus type 2 (PCV2) is the causative agent of postweaning multisystemic wasting syndrome and other PCV2-associated diseases (1, 2). Its epidemic strains continuously evolve, with multiple genotypes co-circulating, and current vaccines use a single antigen, resulting in them having limited protective efficacy (3–5). Porcine circovirus type 3 (PCV3) is an emerging pathogen that is closely associated with porcine dermatitis, nephropathy syndrome, and reproductive disorders (6, 7). PCV2 and PCV3 are widespread in many pig-rearing countries and severely compromise swine health. Co-infection with these two viruses is frequently noted in clinical practice, with a particularly high detection rate of PCV2 in PCV3-positive samples (8–12). No effective bivalent vaccine against PCV2 and PCV3 has been developed globally to date. Multivalent vaccines hold significant promise for the prevention and control of complex and increasingly diverse pathogens.

Nanoparticle antigens exhibit markedly superior immunogenicity compared with conventional protein antigens. Protein nanoparticles are typically formed by the ordered self-assembly of multiple protein subunits into defined spatial structures. Among these, mi3—a sequence derived from the mutated and optimized i301, which originates from the aldolase of the hyperthermophilic bacterium *Thermotoga maritima* (13)—possesses the ability to self-assemble into nanocages composed of 60 mi3 subunits with diameters of approximately 20 nm and a dodecahedral cage-like structure (14). This self-assembly process does not require special conditions, is highly efficient, and can occur in conventional solution environments at room temperature (13); the resulting mi3 nanoparticles are uniform in size and structurally stable (14). SpyCatcher (SC) and SpyTag (ST) domains can rapidly and spontaneously form a covalent isopeptide bond at 4–37 °C and pH 5–8 (15–17). Both domains are small and structurally simple, and they can be genetically fused to two functional proteins, enabling covalent conjugation under mild conditions (14, 18–20). Nanoparticle vaccines based on mi3 and the SpyCatcher/SpyTag system have been developed for pathogens such as severe acute respiratory syndrome coronavirus 2 (SARS-CoV-2) and human immunodeficiency virus (HIV), eliciting robust immune responses in animal models (21–23).

Based on these properties, to construct a highly effective bivalent nanoparticle antigen (PCV2-PCV3 Nano), the Cap protein sequences of prevalent PCV2 and PCV3 strains were analyzed, optimized PCV2 Cap and PCV3 Cap amino acid sequences were obtained, and the SC or ST domain was fused to mi3, PCV2 Cap, or PCV3 Cap. The corresponding genes were codon-optimized, cloned into pET28a(+), and expressed in *E. coli* as fusion proteins. Following purification, PCV2 Cap and PCV3 Cap were covalently displayed on the surface of mi3 nanocages via SC–ST bioconjugation to generate PCV2-PCV3 Nano. This nanoparticle was subsequently used as a vaccine antigen in BALB/c mice, and its immune responses were evaluated and compared with those induced by traditional protein antigens.

## 2 Materials and methods

### 2.1 Animals and ethics statement

Six-week-old female specific pathogen-free (SPF) BALB/c mice were purchased from China Beijing HFK Bioscience Co., Ltd. All of the animal experiments were approved by the Animal Ethics Committee of Hebei Agricultural University (Approval No. 2023159).

### 2.2 Plasmids, cells, and viral strains

The pET28a(+) plasmid for recombinant protein expression and BL21(DE3) *E. coli* competent cells were purchased from Sangon Biotech (Shanghai) Co., Ltd. The PCV2 ND (PCV2b) and PCV2 HB-BD (PCV2d) strains were propagated in PK-15 cells; they were then isolated and preserved at the laboratory. Rabbit antisera against PCV2 and PCV3 were prepared in the laboratory. PCV3 tissue homogenate (10^4.89^ copies/μL) was prepared from mixed tissue samples from porcine lymph nodes, spleens, and lungs that tested positive for PCV3 and negative for other common pathogens via nucleic acid detection. The tissues were ground and homogenized, and the supernatant was collected, filter-sterilized, and used in homogenate preparation.

### 2.3 Design and synthesis of recombinant genes

**Figure 1:**
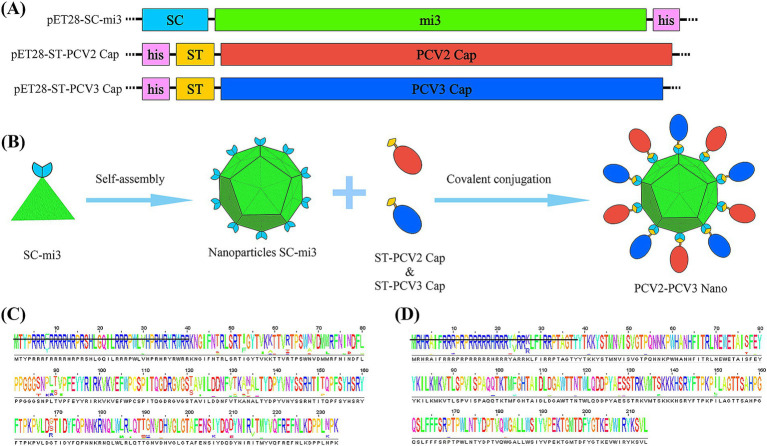
Schematic diagram of the construction strategy for bivalent nanoparticles based on mi3 displaying PCV2 and PCV3 capsid proteins. **(A)** Design of recombinant fusion proteins. **(B)** Schematic illustration of the PCV2-PCV3 nano assembly process. **(C)** Optimized PCV2 Cap amino acid sequence; the deleted N-terminal 40 amino acids are indicated by strikethrough. **(D)** Optimized PCV3 Cap amino acid sequence; the deleted N-terminal 38 amino acids are indicated by strikethrough.

Jalview software was used to optimize each amino acid position of PCV2 Cap and PCV3 Cap according to the principle of maximum occurrence frequency based on the Cap protein amino acid sequences of prevalent PCV2 (71 sequences) and PCV3 (56 sequences) strains deposited in GenBank. The optimized sequences were subsequently modified by deleting the N-terminal 40 amino acids of PCV2 Cap and the N-terminal 38 amino acids of PCV3 Cap. ST was fused to the N-terminus of PCV2 Cap and PCV3 Cap, and SC was fused to the N-terminus of mi3, according to the construction strategy (Figures 1A,B). A His tag was added for affinity chromatography purification. The fusion sequences were codon-optimized for *E. coli*, synthesized by Sangon Biotech (Shanghai) Co., Ltd., and inserted into the pET28a(+) vector to generate recombinant expression plasmids.

### 2.4 Protein expression and purification

The recombinant plasmids were individually transformed into BL21(DE3) competent cells by heat shock, plated on kanamycin-containing plates, and cultured overnight at 37 °C. Single colonies were selected and identified by polymerase chain reaction (PCR) using T7-F/R primers. Positive clones, designated as engineered strains, were inoculated into LB liquid medium and cultured at 37 °C with shaking at 200 rpm for 10 h as seed cultures. Subsequently, 2% of the seed culture was transferred into 200 mL of LB medium and cultured at 37 °C with shaking at 200 rpm for 4 h. Isopropyl-𝛽-D-1-thiogalactopyranoside was added to a final concentration of 0.5 mmol/L, and induction was continued at 25 °C with shaking at 200 rpm for 10 h. The fermentation broth was centrifuged to collect the bacterial pellet, which was resuspended in 25 mL of equilibration buffer (20 mM Tris–HCl, 10 mM imidazole, 0.5 M NaCl, pH 8.0). Following ultrasonication, the lysate was centrifuged at 12,000 × g for 15 min at 4 °C, and the supernatant was collected. Proteins were purified using Ni–agarose resin (Kangwei Century, Beijing, China). Endotoxins were removed with a Protein Endotoxin Removal Kit (Beyotime, Shanghai, China), and the residual endotoxin level was measured using a Chromogenic LAL Endotoxin Assay Kit (Beyotime, Shanghai, China). Protein samples were analyzed by sodium dodecyl sulfate-polyacrylamide gel electrophoresis (SDS-PAGE), and purified antigen proteins were validated by western blotting.

### 2.5 Assembly of bivalent PCV2 and PCV3 nanoparticles

Purified proteins were dialyzed against assembly buffer (20 mM Tris–HCl, pH 8.0). The pre-assembled SC-mi3 nanoparticle solution was mixed with ST-PCV2 Cap or ST-PCV3 Cap solutions at different molar ratios and then incubated at 4 °C for 16–20 h with occasional shaking. Conjugation efficiency was evaluated by SDS-PAGE to determine the optimal molar ratio. ST-PCV2 Cap and ST-PCV3 Cap were first mixed at the optimal molar ratio and then conjugated with the SC-mi3 nanoparticle solution under the same conditions to obtain the bivalent PCV2 and PCV3 nanoparticle (PCV2-PCV3 Nano), which was then validated by SDS-PAGE.

### 2.6 Characterization of nanoparticles

SC-mi3 nanoparticles and PCV2-PCV3 Nano solutions were appropriately diluted, adsorbed onto copper grids, negatively stained with 2% phosphotungstic acid, and then observed by transmission electron microscopy (TEM) (JEOL, Japan). Dynamic light scattering (DLS) using a Zetasizer Nano ZS90 (Malvern, UK) was conducted to determine particle size distribution and zeta potential.

### 2.7 Animal immunization and challenge

Equimolar mixtures of ST-PCV2 Cap and ST-PCV3 Cap and PCV2-PCV3 Nano solutions were appropriately diluted, mixed with ISA 206 VG adjuvant (Seppic, France) at a 1:1 (v/v) ratio, and emulsified as vaccines. Six-week-old female SPF BALB/c mice (*n* = 40) were randomly divided into four groups, namely, (1) the PBS group, (2) the PCV2+PCV3 group (equimolar mixture of ST-PCV2 Cap and ST-PCV3 Cap), (3) the PCV2-PCV3 Nano group, and (4) the PCV2 Com group (commercial PCV2 vaccine). Mice were immunized intramuscularly with 20 μg of antigen on day 0, and they received a booster immunization on day 14. On day 28, five mice from each group were euthanized for immunological assays. The remaining mice—excluding the PCV2 Com group—were intraperitoneally injected with 0.1 mL of PCV2 HB-BD (10^4.6^ TCID_50_) and 0.1 mL of PCV3 tissue homogenate. Mice in the PCV2 Com group were injected only with PCV2 HB-BD. Body weight was measured every 2 days after challenge, and all of the mice were euthanized on day 42. In this study, all mice were humanely euthanized using the following procedure: the mice were placed in a dedicated sealed chamber and administered a gradual infusion of carbon dioxide (CO_2_) for 3–5 min, after which cervical dislocation was performed to confirm death.

In the cross-protection study against different PCV2 genotypes, age-matched mice (*n* = 15) were randomly divided into three groups, namely, the control group (challenged with PCV2 ND), the PCV2b group (challenged with PCV2 ND), and the PCV2d group (challenged with PCV2 HB-BD). Mice—excluding the control group—were immunized once with PCV2-PCV3 Nano. Blood was collected for neutralizing antibody detection on day 21, and mice were challenged with the same dose. Blood was collected for viral load detection on day 7 post-challenge, and mice were then euthanized.

### 2.8 Determination of humoral and cellular immune responses

Enzyme-linked immunosorbent assay (ELISA): Blood was collected via the submandibular vein on days 0, 7, 14, 21, and 28 after the initial immunization, and serum was separated, diluted at a ratio of 1:50, and further subjected to 2-fold serial dilutions to 50 × 2^11^. PCV2-specific IgG antibodies were detected with a Porcine Circovirus Type 2 Antibody Detection Kit (Shenzhen Lvshiyuan Biotechnology Co., Ltd., Shenzhen, China), with the secondary antibody replaced by Goat Anti-Mouse IgG/HRP (Solarbio, Beijing, China) diluted at a ratio of 1:5000, with all other procedures performed according to the manufacturer’s instructions. GST-PCV3 Cap protein expressed and purified from the laboratory-constructed BL21-pGEX-4 T-1-PCV3/Cap strain was used as the coating antigen to detect PCV3-specific IgG antibodies by ELISA. The results were established as follows: a sample was considered positive when the OD_450_ of the test serum/OD_450_ of the negative serum (P/N) was ≥2.1. The highest serum dilution with a P/N ≥ 2.1 was set as the antibody titer.

Neutralization tests: Serum collected on day 28 was serially diluted 2-fold with Dulbecco’s modified eagle’s medium (Wisent, Nanjing, China) containing 10% fetal bovine serum (Opcel, Inner Mongolia, China). Subsequently, 50 μL of diluted serum was mixed with 50 μL of PCV2 HB-BD containing 100 TCID_50_ and incubated at 37 °C for 1 h. The mixture was inoculated into freshly passaged PK-15 cells (96-well plates) and cultured at 37 °C for 72 h. Cells were fixed with 4% paraformaldehyde, and viral infection was detected by indirect immunofluorescence assay. The neutralizing antibody titer was defined as the highest serum dilution that reduced the number of fluorescence-positive cells by 80% compared with the virus control group.

IFN-γ and IL-4 detection: On day 28, spleens were collected from mice, and splenocyte suspensions were prepared. The cells were diluted with 1640 medium (Wisent, Nanjing, China) to 2 × 10^6^ cells/well (24-well plates) and stimulated with 10 μg/mL ST-PCV2 Cap or concanavalin A (ConA). After incubation at 37 °C for 72 h, the supernatant was collected by centrifugation. IFN-γ and IL-4 levels were measured using mouse IFN-γ/IL-4 ELISA kits (Jiangsu Enzyme Exemption Industry Co., Ltd., Jiangsu, China).

### 2.9 Evaluation of immune protection

Mice were weighed on days 0, 2, 4, 6, 8, 10, 12, and 14 post-challenge, and the relative weight gain rate was calculated as follows:

$$\begin{array}{l} \text{Relative weight gain rate}\;(\%)\\ =(\begin{array}{l} \text{Body weight}\;\mathrm{on}\;\mathrm{day}\;14\;\text{post}-\text{challenge}\\ –\text{Body weight}\;\mathrm{on}\;\text{the}\;\mathrm{day}\;\text{of challenge} \end{array})/\\ \text{Body weight}\;\mathrm{on}\;\text{the}\;\mathrm{day}\;\text{of challenge}\times 100\%. \end{array}$$

Serum was collected on days 7 and 14 post-challenge. PCV2 and PCV3 viral loads in serum were measured by SYBR Green quantitative PCR using GS AntiQ qPCR SYBR Green Fast Mix (Genesand Biotech Co., Ltd., Beijing, China). The primer sequences are listed in Table 1.

**Table 1:** Primers used in this study.

Name	Sequences (5′-3′)
T7-F	TAATACGACTCACTATAGGG
T7-R	GCTAGTTATTGCTCAGCGG
PCV2-F	TCTGAATTGTACATACATGG
PCV2-R	CCCTTTGAATACTACAGAA
PCV3-F	AGACGACGACGCCACAGGAG
PCV3-R	GGGCTT GTTATTCTGAGGGGTTCC

T7-F/R was used to identify positive clones of the engineered bacteria. PCV2-F/R and PCV3-F/R were used to detect the viral loads of PCV2 and PCV3 in serum, respectively.

### 2.10 Statistical analysis

All of the data were statistically analyzed using GraphPad Prism 10. Comparisons between groups were performed using an unpaired *t*-test. Significance was defined as follows: *p* < 0.001 (***), *p* < 0.01 (**), *p* < 0.05 (*), *p* ≥ 0.05 (ns).

## 3 Results

### 3.1 Design of self-assembling bivalent nanoparticles based on mi3 displaying PCV2 and PCV3 capsid proteins

Construction was performed according to the strategy shown in Figures 1A,B in order to prepare self-assembling bivalent nanoparticles based on mi3 displaying PCV2 and PCV3 capsid proteins. The optimized PCV2 Cap and PCV3 Cap amino acid sequences with NLS deletion are shown in Figures 1C,D.

### 3.2 Construction of engineered *Escherichia coli* strains and protein purification

**Figure 2:**
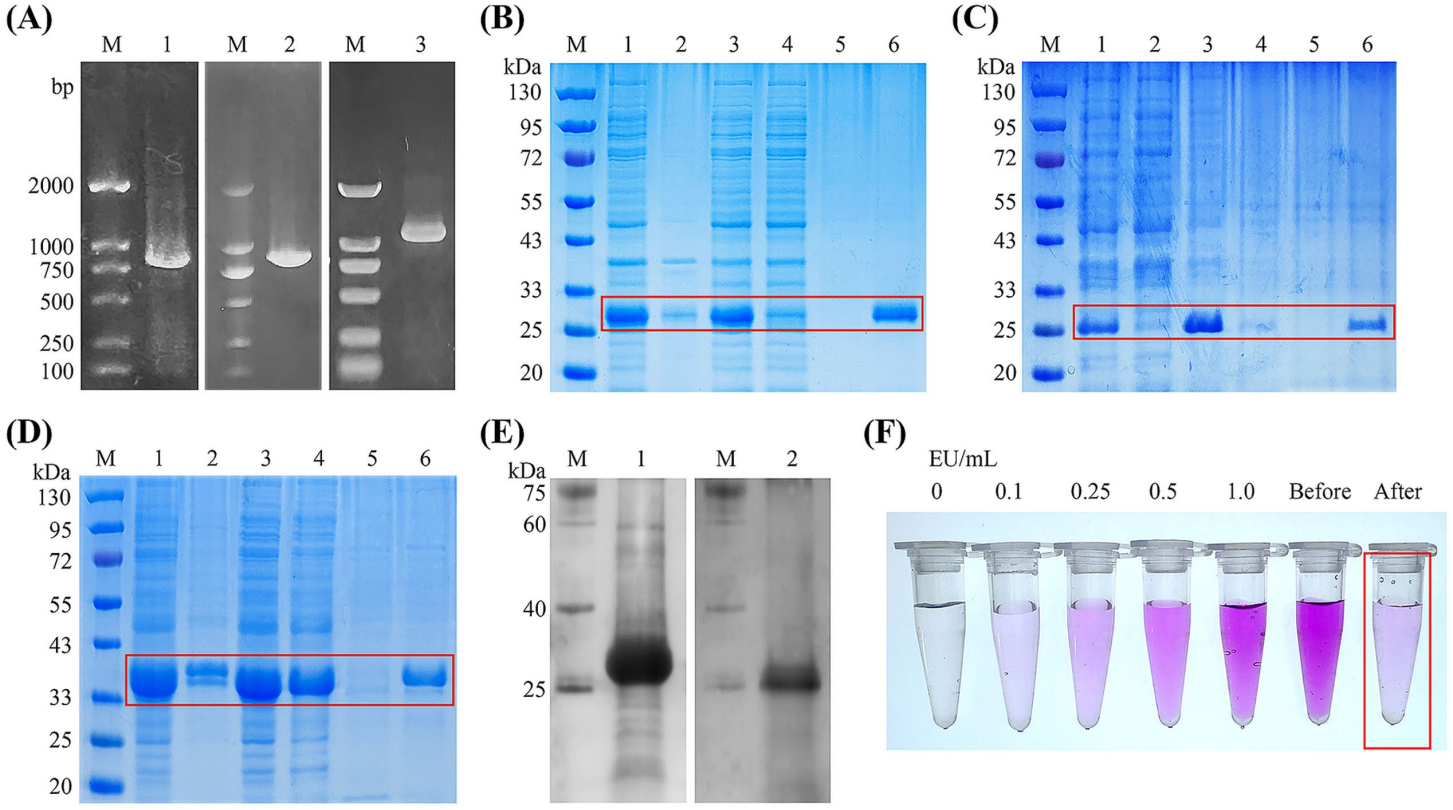
Construction of engineered *E. coli* strains and protein purification. **(A)** PCR identification of positive *E. coli* clones. Lanes 1–3 correspond to BL21-pET28-ST-PCV2 Cap, BL21-pET28-ST-PCV3 Cap, and BL21-pET28-SC-mi3, respectively. **(B–D)** Expression and purification of recombinant proteins ST-PCV2 Cap, ST-PCV3 Cap, and SC-mi3, respectively. Lanes 1–6 represent whole bacterial lysate, pellet after sonication, supernatant after sonication, Ni column flow-through, wash fraction, and purified protein, respectively. **(E)** Western blot validation of antigen proteins. Lanes 1, 2 correspond to ST-PCV2 Cap and ST-PCV3 Cap, respectively. **(F)** Endotoxin removal from recombinant protein solutions. Color intensity represents endotoxin content; numbers indicate endotoxin content (EU/mL).

The three recombinant expression plasmids constructed according to the strategy in Figure 1A were designated pET28-ST-PCV2 Cap, pET28-ST-PCV3 Cap, and pET28-SC-mi3. After transformation into BL21(DE3), single colonies were selected and identified by PCR. Positive clones were obtained as engineered strains (Figure 2A) and designated BL21-pET28-ST-PCV2 Cap, BL21-pET28-ST-PCV3 Cap, and BL21-pET28-SC-mi3. SDS-PAGE analysis showed that all three recombinant of the proteins were present in the soluble fraction of the cell lysate. After Ni affinity chromatography, purified ST-PCV2 Cap (26.0 kDa), ST-PCV3 Cap (23.9 kDa), and SC-mi3 (34.2 kDa) were obtained (Figures 2B–D). Western blot analysis using rabbit antisera confirmed the antigenicity of purified ST-PCV2 Cap and ST-PCV3 Cap (Figure 2E). After endotoxin removal, the endotoxin content in all of the protein solutions was below 0.25 EU/mL (Figure 2F), eliminating potential interference from endotoxins in subsequent animal experiments.

### 3.3 Assembly and conjugation of PCV2-PCV3 nano

**Figure 3:**
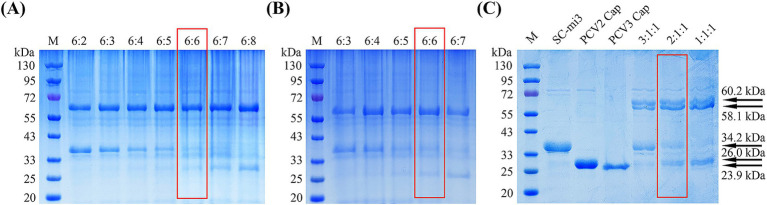
SDS-PAGE validation of PCV2-PCV3 nano assembly and conjugation products. **(A,B)** Optimization of the molar ratio for conjugation of SC-mi3 with ST-PCV2 Cap and ST-PCV3 Cap, respectively. Red boxes indicate lanes corresponding to the optimal molar ratio. **(C)** Optimal molar ratio for the conjugation of SC-mi3, ST-PCV2 Cap, and ST-PCV3 Cap.

The self-assembled SC-mi3 nanoparticles were conjugated with ST-PCV2 Cap or ST-PCV3 Cap. SDS-PAGE analysis revealed that the molecular weights of the conjugates were 60.2 kDa and 58.1 kDa, respectively. The optimal molar ratio for both conjugations was 1:1 (Figures 3A,B, red boxes), indicating that SC and ST form an equimolar covalent bond. To display equal amounts of PCV2 Cap and PCV3 Cap on the surface of the bivalent nanoparticles, conjugation was performed using an SC-mi3: ST-PCV2 Cap: ST-PCV3 Cap molar ratio of 2:1:1. The SDS-PAGE result was consistent with expectations (Figure 3C), thus confirming the successful generation of PCV2-PCV3 Nano.

### 3.4 Characterization of nanoparticles

**Figure 4:**
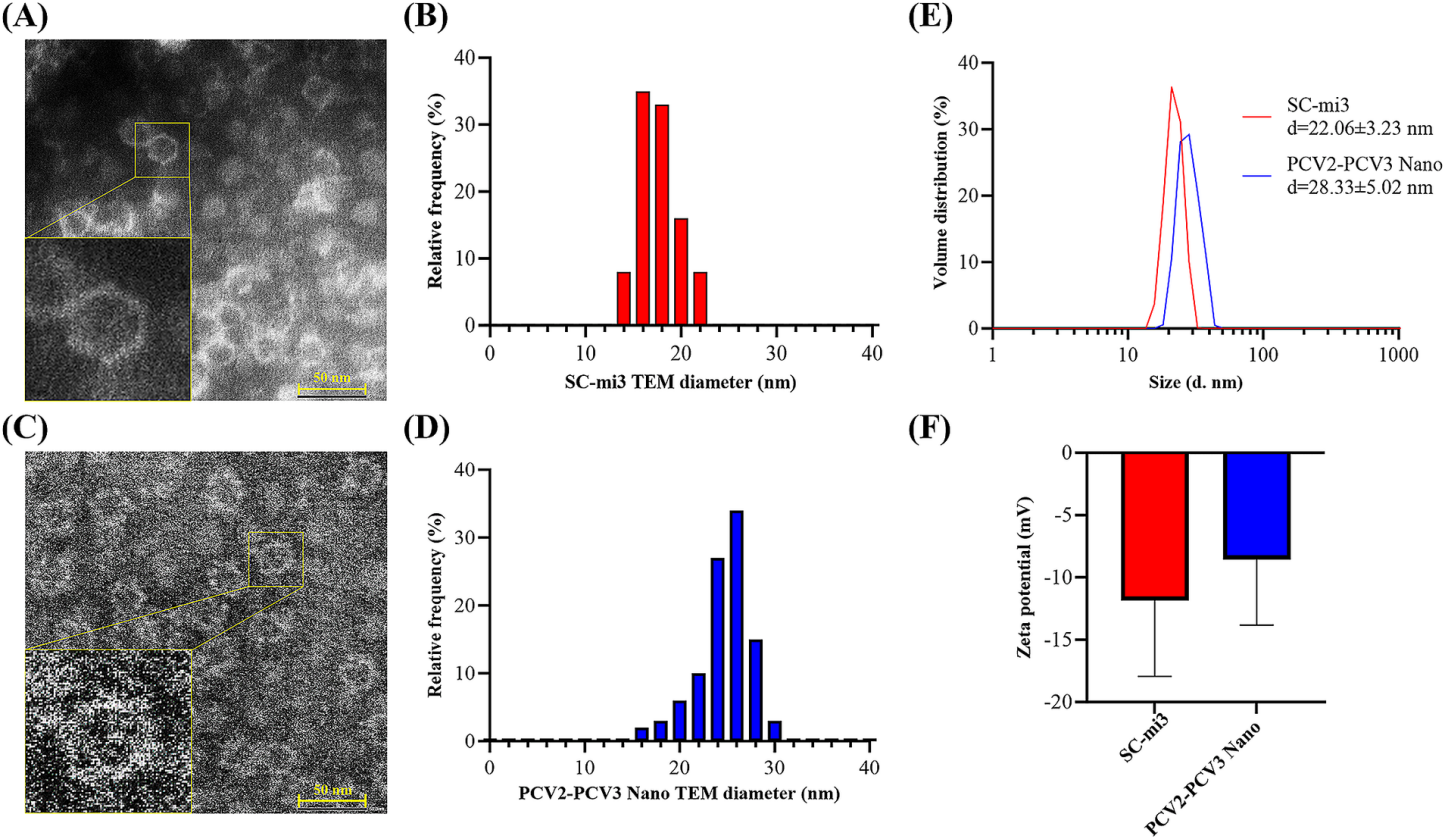
Characterization of mi3-based nanoparticles. **(A,B)** TEM observation and particle size analysis of SC-mi3 nanoparticles. **(C,D)** TEM observation and particle size analysis of PCV2-PCV3 Nano. **(E)** DLS analysis of nanoparticle size distribution. **(F)** Zeta potential of nanoparticle solutions.

TEM observation revealed that both SC-mi3 nanoparticles and PCV2-PCV3 Nano exhibited polyhedral spherical morphology with uniform particle size and no visible impurities. The morphology of PCV2-PCV3 Nano was slightly less compact than that of SC-mi3 (Figures 4A,B). Size analysis showed that SC-mi3 nanoparticles had a diameter of approximately 20 nm, whereas PCV2-PCV3 Nano exhibited a significantly larger diameter (Figures 4C,D). DLS measurement further confirmed that the particle size of SC-mi3 was 22.06 ± 3.23 nm, and that of PCV2-PCV3 Nano was 28.33 ± 5.02 nm (Figure 4E). Zeta potential analysis showed that PCV2-PCV3 Nano had a value of ˗8.57 mV (Figure 4F), indicating that the solution system remained stable.

### 3.5 Humoral and cellular immune responses

**Figure 5:**
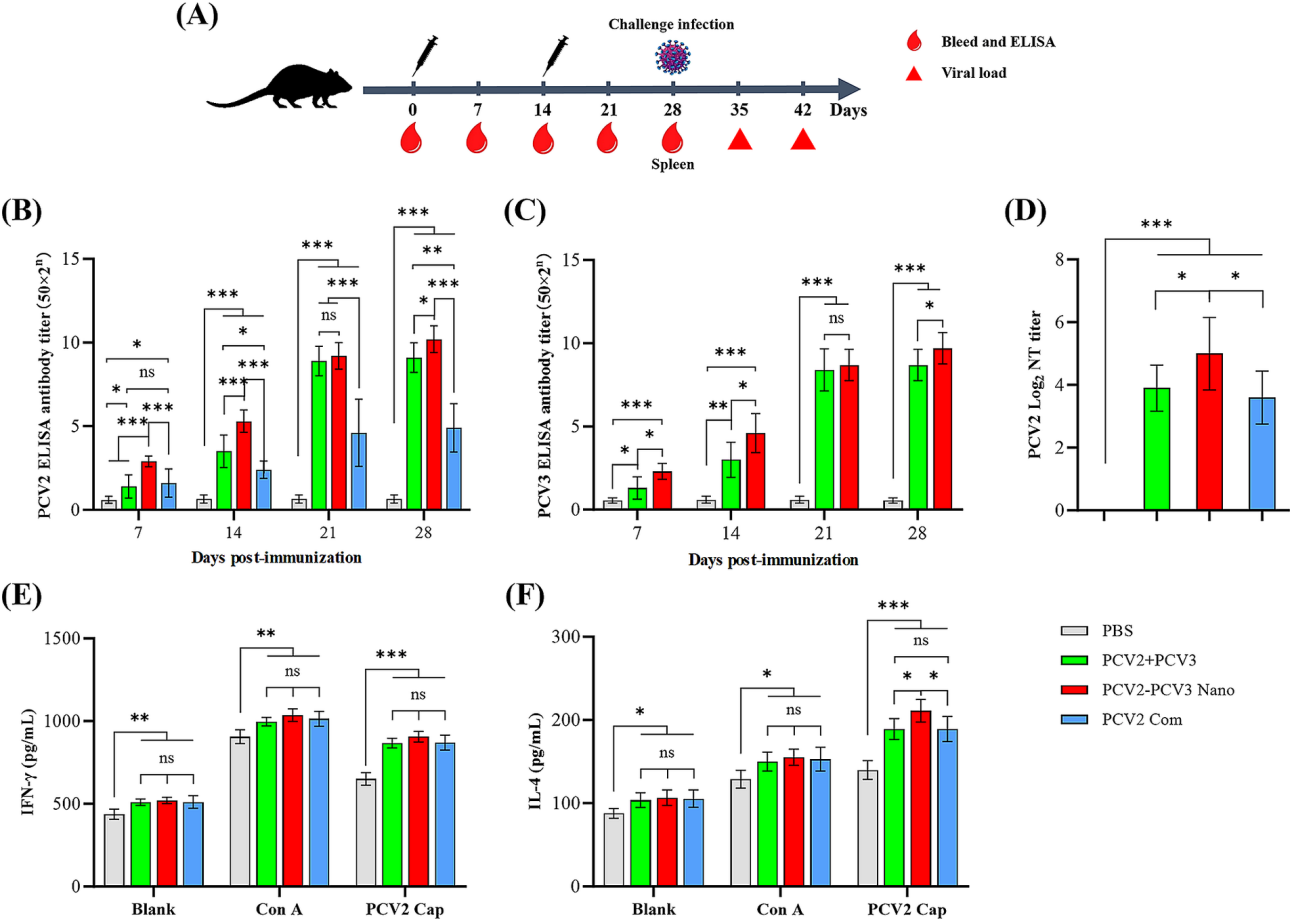
Immune responses in mice. **(A)** Schematic timeline of the immunization and challenge experiments. **(B,C)** PCV2 and PCV3 ELISA antibody titers at different time points. **(D)** PCV2 neutralizing antibody titers in each group on day 28. **(E,F)** IFN-γ and IL-4 concentrations (pg/mL) in the supernatant of splenocytes stimulated with different agents. Data are presented as mean ± SD (*n* = 10). *p < 0.05, **p < 0.01, ***p < 0.001, ns indicates no significance.

The animal experimental scheme is shown in Figure 5A. ELISA results showed that PCV2-specific IgG antibodies were detected from day 7 post-immunization and continuously increased up to day 28. Antibody levels followed the order PCV2-PCV3 Nano > PCV2+PCV3 > PCV2 Com > PBS group. The antibody titer in the PCV2-PCV3 Nano group reached 50 × 2^10.2^ on day 28, which was more than twice that of the PCV2+PCV3 group (50 × 2^9.1^) (Figure 5B). The levels of PCV3-specific IgG antibodies were lower than those of PCV2, but the trend was consistent. The antibody titer in the PCV2-PCV3 Nano group reached 50 × 2^9.7^ on day 28, and it was also approximately twice that of the PCV2+PCV3 group (50 × 2^8.7^) (Figure 5C). PCV2-neutralizing antibodies were detected in all of the vaccinated groups on day 28, with the PCV2-PCV3 Nano group exhibiting the highest neutralizing titer (2^5.0^) (Figure 5D). IFN-γ and IL-4 detection showed that cytokine levels in all of the vaccinated groups were higher than those in the PBS group. However, except for a slightly higher IL-4 concentration in the PCV2-PCV3 Nano group, no significant differences were observed among the vaccinated groups (Figures 5E,F). These results indicate that PCV2-PCV3 Nano markedly enhances humoral immunity compared with conventional antigens, whereas its effect on cellular immunity is limited.

### 3.6 Challenge protection efficacy

**Figure 6:**
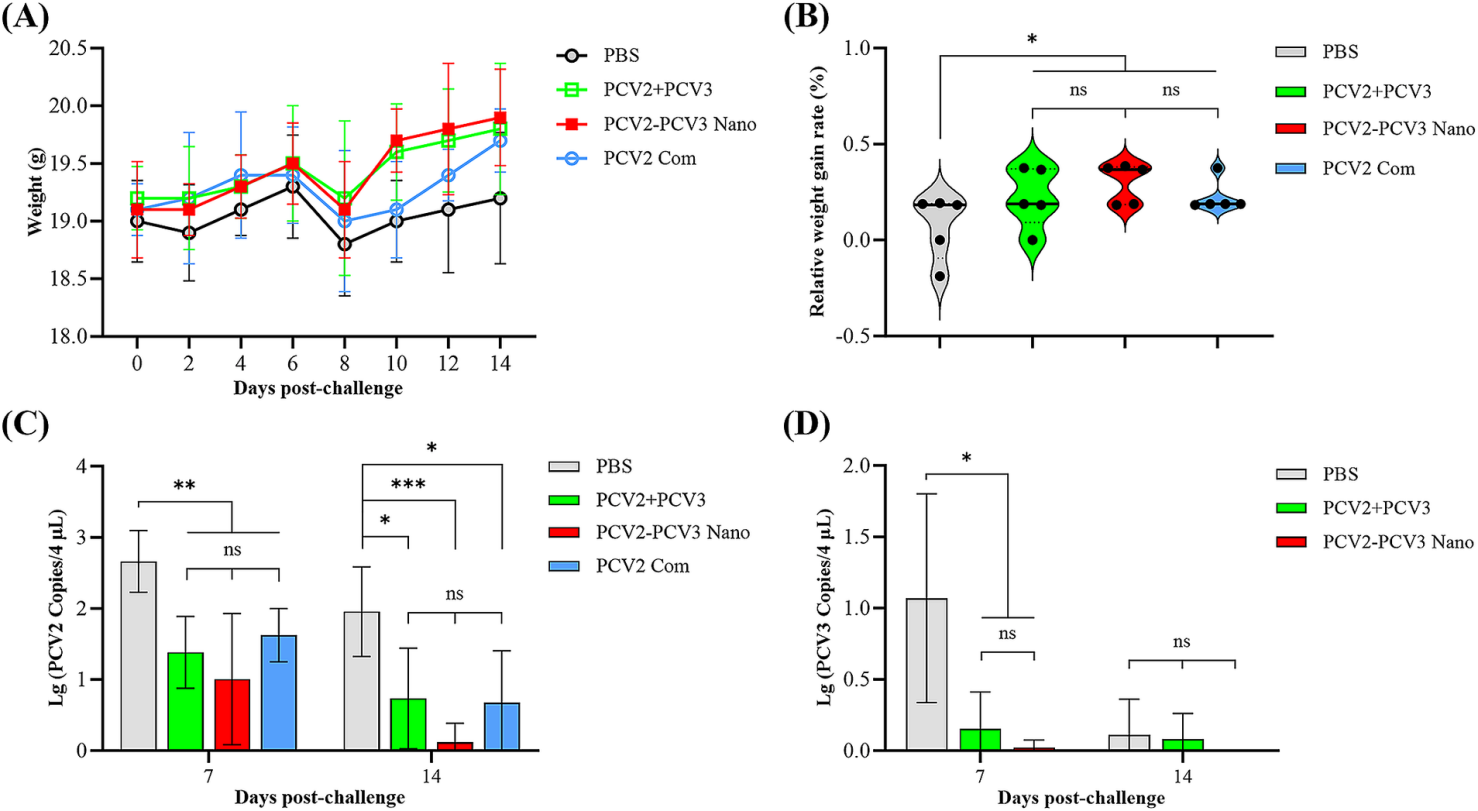
Challenge protection efficacy. **(A)** Body weight changes in mice after challenge with PCV2 and PCV3. **(B)** Relative weight gain rates of mice in each group on day 14 post-challenge. **(C,D)** PCV2 and PCV3 viral loads in serum on days 7 and 14 post-challenge. Data are presented as mean ± SD (*n* = 5). *p < 0.05, **p < 0.01, ***p < 0.001, ns indicates no significance.

By day 14 post-challenge, mice in all of the vaccinated groups had gained weight, with the PCV2-PCV3 Nano group showing the greatest increase (Figure 6A), although differences among groups were not significant. Body weight changes in the PBS group were minimal, and some individuals exhibited weight loss (Figures 6A,B).

Serum PCV2 viral loads in all of the vaccinated groups were significantly lower than those in the PBS group on day 7 post-challenge, with no significant differences among vaccinated groups. PCV2 nucleic acid was not detected in some samples from the PCV2-PCV3 Nano group. PCV2 viral loads in all of the groups further decreased on day 14 post-challenge, and PCV2 nucleic acid was not detected in 4/5 of the samples from the PCV2-PCV3 Nano group (Figure 6C). Regarding PCV3 viral loads, the PCV2-PCV3 Nano and PCV2+PCV3 groups exhibited significantly lower levels than the PBS group on day 7 post-challenge, with most samples negative for PCV3 nucleic acid. PCV3 was almost completely cleared in all of the groups on day 14 post-challenge (Figure 6D). These results demonstrate that immunization with PCV2-PCV3 Nano provides protective efficacy against both PCV2 and PCV3.

### 3.7 Cross-protective efficacy of PCV2-PCV3 nano against PCV2b and PCV2d

**Figure 7:**
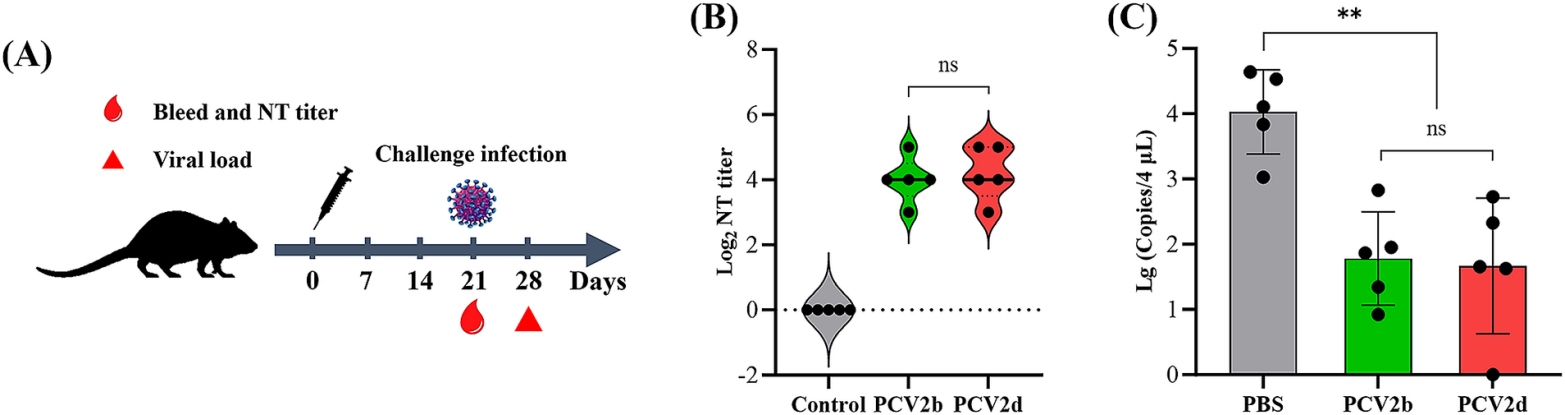
Cross-protective efficacy of PCV2-PCV3 nano against PCV2b and PCV2d. **(A)** Schematic timeline of the immunization and challenge experiments. **(B)** Neutralizing antibody titers against PCV2b and PCV2d on day 21. **(C)** PCV2 viral loads in serum on day 7 post-challenge. Data are presented as mean ± SD (*n* = 5). **p < 0.01, ns indicates no significance.

The experimental scheme for evaluating the challenge protection efficacy of PCV2-PCV3 Nano against PCV2b and PCV2d in mice is shown in Figure 7A. Neutralizing antibody detection revealed that serum from immunized mice exhibited neutralizing activity against both PCV2b and PCV2d, with no significant difference between the two genotypes (Figure 7B). Serum viral loads in both the PCV2b and PCV2d groups were lower than those in the control group on day 7 post-challenge, with no significant difference between the two challenge groups (Figure 7C). These results indicate that immunization with PCV2-PCV3 Nano confers cross-protective efficacy against both PCV2b and PCV2d genotypes.

## 4 Discussion

mi3 nanoparticles are formed by the self-assembly of 60 mi3 subunits into a hollow, porous cage-like structure, also referred to as a nanocage (14). In this study, TEM observation revealed nanoparticles with a light periphery and a dark center, consistent with this structural feature. This cage-like structure has a large specific surface area, allowing antigens to be displayed on the surface or encapsulated within the structure. The resulting PCV2-PCV3 Nano exhibits a potent self-adjuvant effect. On the one hand, the nanoparticles are optimal for lymphatic drainage, allowing passive entry into lymphatic vessels through tissue gaps and direct transport to lymph nodes; on the other hand, surface-displayed antigens facilitate antigen presentation and rapid immune responses, while internal encapsulation enhances stability and provides a sustained-release effect, thereby eliciting long-lasting immune stimulation (24).

Every mi3 monomer is fused with one SC domain. Theoretically, a single mi3 nanocage can conjugate 60 antigen molecules fused with ST. In this study, the observed conjugation molar ratio of SC-mi3: ST-PCV2 Cap: ST-PCV3 Cap = 2: 1: 1 (Figure 3C) was consistent with this theoretical value, which may be attributed to the relatively small molecular sizes of PCV2 Cap and PCV3 Cap. When conjugating larger antigen molecules, steric hindrance may alter the conjugation ratio, necessitating optimization for different antigens. The molar ratio of ST-PCV2 Cap to ST-PCV3 Cap bound to the SC-mi3 nanocage was controlled at 1:1 in this study in order to ensure a balanced immune response against PCV2 and PCV3 after immunization.

No commercial PCV3 vaccine is currently available. Although PCV2 recombinant subunit vaccines have been commercialized and can form VLPs (25), most products exhibit low VLP assembly efficiency, are prone to disassembly, or fail to assemble, thereby affecting vaccine immunogenicity. mi3 self-assembly is highly efficient and straightforward to perform. In this study, the purified SC-mi3 solution directly assembled into stable nanoparticles with high efficiency. Storage conditions, such as refrigeration, freeze–thaw cycles, heating, and lyophilization, did not affect its assembly, consistent with prior reports (14); it can even remain stable at 80 °C in 6.7 M guanidine hydrochloride (13). The conjugation efficiency of SC and ST was also extremely high, requiring no special conditions and remaining unaffected even in high concentrations of urea. The resulting covalent isopeptide bond is structurally robust and does not dissociate during subsequent processing. In this study, the NLS was deleted from the PCV2 Cap and PCV3 Cap sequences (26, 27). The NLS is a key structural element for the self-assembly of Cap proteins into VLPs (27, 28). However, to avoid interference from VLP self-assembly with nanoparticle conjugation, the NLS was removed, thereby facilitating antigen display on the mi3 nanocage surface.

Further, optimization of the PCV2 Cap and PCV3 Cap amino acid sequences considered the sequence characteristics of prevalent strains, which is expected to induce broader immune protection. Because the currently predominant PCV2 strains are mainly of the PCV2b and PCV2d genotypes, and the PCV3 genome has not undergone significant variation since its initial report, only PCV2b, PCV2d, and PCV3 were selected as challenge strains in this study to evaluate cross-protective efficacy. By efficiently conjugating optimized PCV2 Cap and PCV3 Cap to highly stable mi3 nanocages via the SC–ST system, this study represents an advancement over traditional subunit vaccines, significantly enhancing immunogenicity and providing a new approach for the development of porcine circovirus vaccines.

In this study, the GST-PCV3 Cap protein expressed from the laboratory-constructed BL21-pGEX-4T-1-PCV3/Cap strain was used as the coating antigen for ELISA, thereby avoiding interference from tag proteins. In addition, because the isolation and culture of PCV3 remain challenging (29–32), PCV3-neutralizing antibodies were not evaluated in this study, and the challenge experiment was performed using tissue homogenate prepared from PCV3-positive pathological material. The replication efficiency of this tissue-derived virus in mice is unclear, and the related results may therefore have certain limitations. However, serum from PBS-challenged mice contained low levels of PCV3 nucleic acid on day 7 post-challenge, whereas levels in the immunized groups were lower or undetectable (Figure 6D). This finding indicates that the immune response accelerated PCV3 clearance, suggesting that PCV2-PCV3 Nano confers protective efficacy against PCV3 infection. In the cross immune protection experiment, the control group was only challenged with PCV2 ND because PCV2 HB-BD had been used in a prior animal experiment and its challenge effectiveness had been demonstrated.

Analysis of ELISA antibody trends revealed that, compared with the PCV2+PCV3 group, antibody levels in the PCV2-PCV3 Nano group continued to increase after day 21. This trend suggests that antibody levels would have further increased on day 28 and beyond, indicating a longer duration of immunity conferred by PCV2-PCV3 Nano. This observation is consistent with the self-adjuvant effect of the PCV2-PCV3 Nano cage structure, demonstrating that the mi3 nanocage not only induces rapid immune responses but also provides a sustained-release effect, thereby enhancing both the magnitude and duration of humoral immunity induced by conventional vaccines. However, no significant differences in IFN-γ and IL-4 levels were observed among vaccinated groups in this study, suggesting that the mi3 nanoparticle has limited capacity to enhance cellular immunity. In summary, PCV2-PCV3 Nano exhibits significant advantages in enhancing humoral immunity and immune protection.

The general decrease in mouse body weight observed on day 8 post-challenge (Figure 6A) may be attributed to stress induced by submandibular vein blood collection on day 7. Nevertheless, the overall trend in body weight changes among groups remained consistent, still demonstrating the immune protection advantage of PCV2-PCV3 Nano.

Further, although a mouse model for PCV immune challenge has been established (33), the absence of immunogenicity evaluation in the target species (pigs) represents a limitation of this study. However, the mouse model sufficiently demonstrates the superiority of the mi3 nanocage-based PCV2-PCV3 Nano antigen in inducing immune responses, thereby achieving the objectives of this study. Based on the current data (Figures 5B,C), it is inferred that PCV2-PCV3 Nano has advantages over conventional antigens in terms of the durability of the immune response. The long-term immune response elicited by this nanoparticle in the target animal (i.e., swine) will be further investigated in future studies.

Finally, the self-assembly property of the mi3 nanocage and the SC–ST conjugation system will contribute to advancing the development of nanoparticle vaccines, particularly for antigens that cannot self-assemble into VLPs, thereby enabling the nano-enhancement of subunit vaccines. Further, this platform supports the development of multivalent vaccines by displaying multiple antigens on a single nanocage, providing technical support for multivalent vaccine research and development.

## 5 Conclusion

In this study, a self-assembling nanoparticle, PCV2-PCV3 Nano, was successfully constructed. This nanoparticle was generated by conjugating PCV2 Cap and PCV3 Cap to mi3 nanocages via the SC–ST system. The nanoparticle exhibited uniform particle size and structural stability. Immunization of BALB/c mice demonstrated that PCV2-PCV3 Nano significantly induced higher antibody levels and conferred immune protection against PCV2b, PCV2d, and PCV3 infections compared with conventional antigens and commercial PCV2 vaccines.

## Data Availability

The raw data supporting the conclusions of this article will be made available by the authors, without undue reservation.
