# Supplementary material for: Construction and immunogenicity evaluation of a bivalent nanoparticle based on mi3 displaying porcine circovirus type 2 and type 3 capsid proteins
Source: Front Vet Sci. 2026 Jun 5;13:1862938. doi: 10.3389/fvets.2026.1862938 (PMC13280793; doi:10.3389/fvets.2026.1862938)
Supplement: Supplementary file 1 [file Image_1.PDF]

*Supplementary Material*

# 1 Supplementary Figures

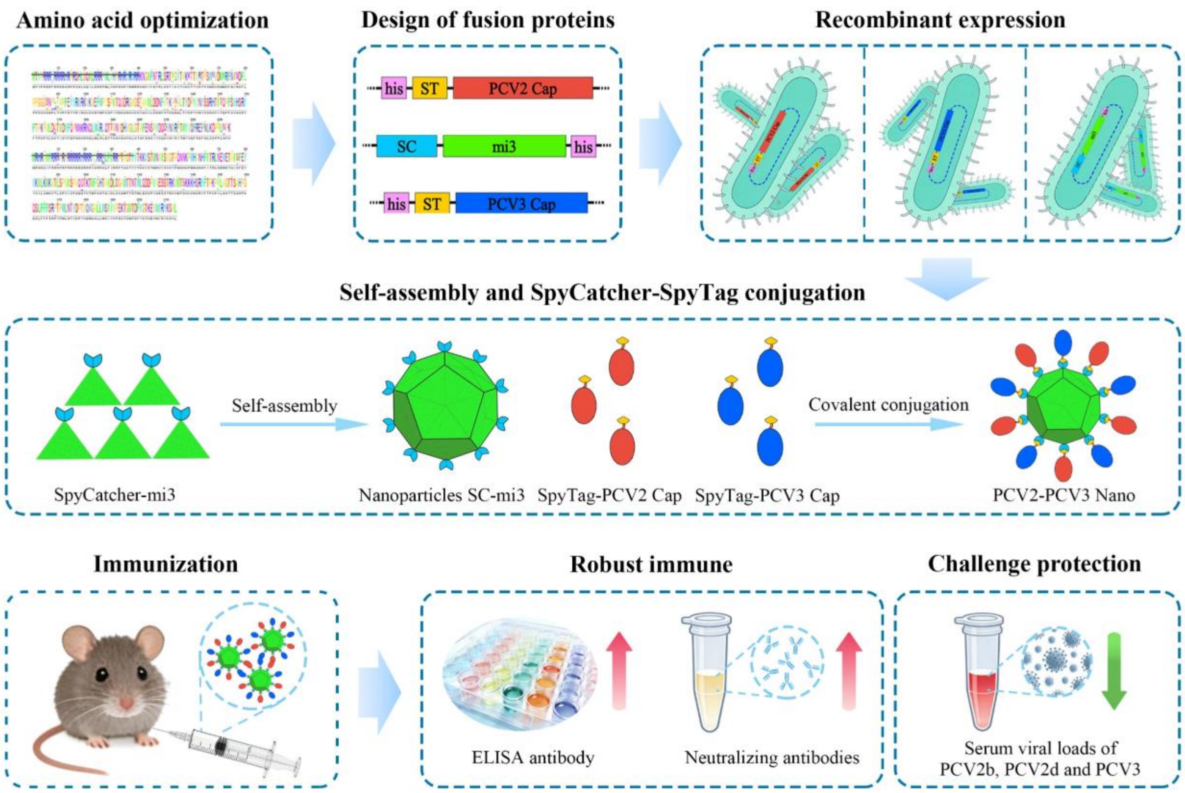


**Supplementary Figure 1.** Graphical Abstract.

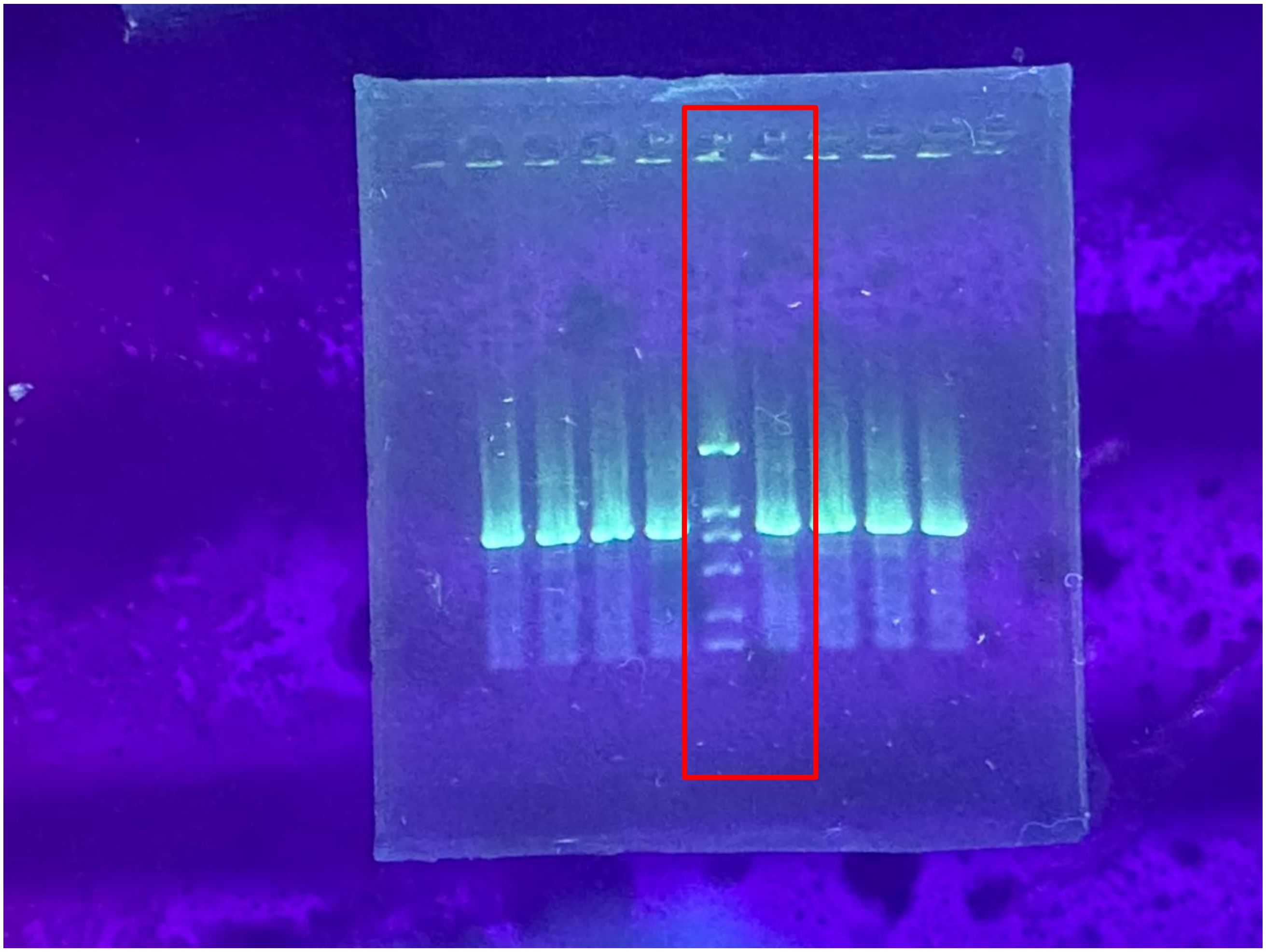

**Supplementary Figure 2.** PCR identification of positive *E. coli* clones of BL21-pET28-ST-PCV2 Cap (Original image).

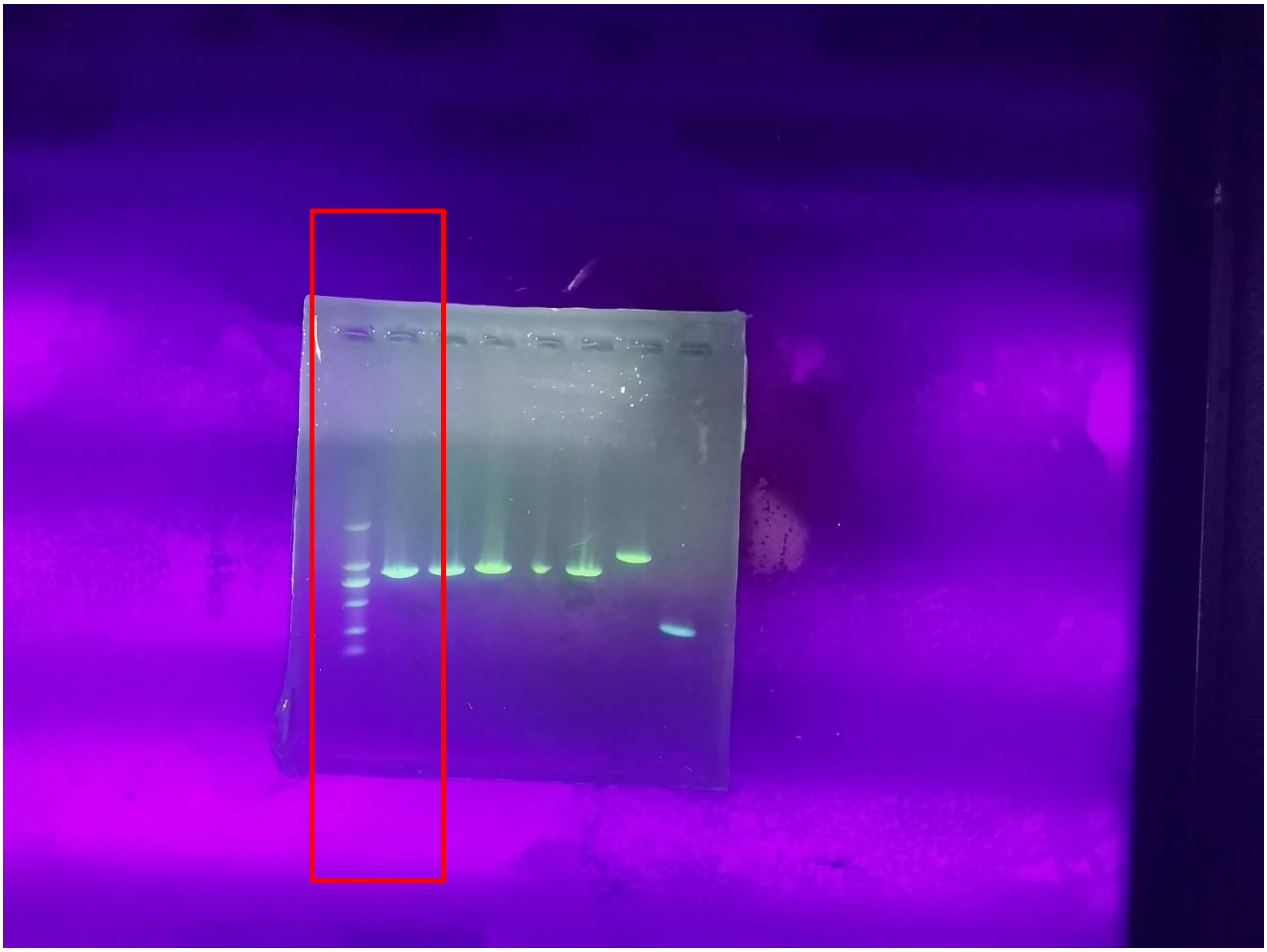

**Supplementary Figure 3.** PCR identification of positive *E. coli* clones of BL21-pET28-ST-PCV3 Cap (Original image).

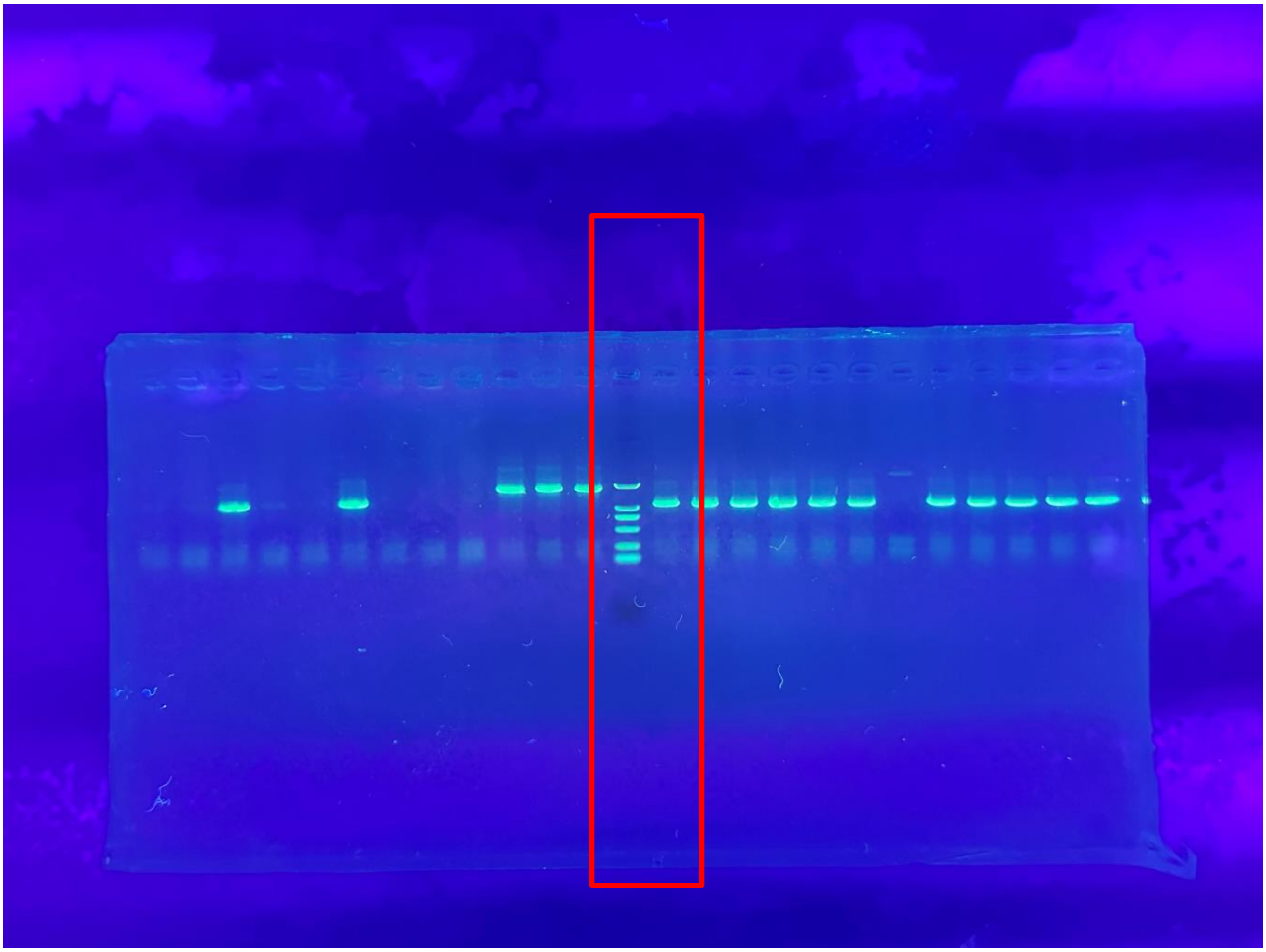

**Supplementary Figure 4.** PCR identification of positive *E. coli* clones of BL21-pET28-SC-mi3 (Original image).

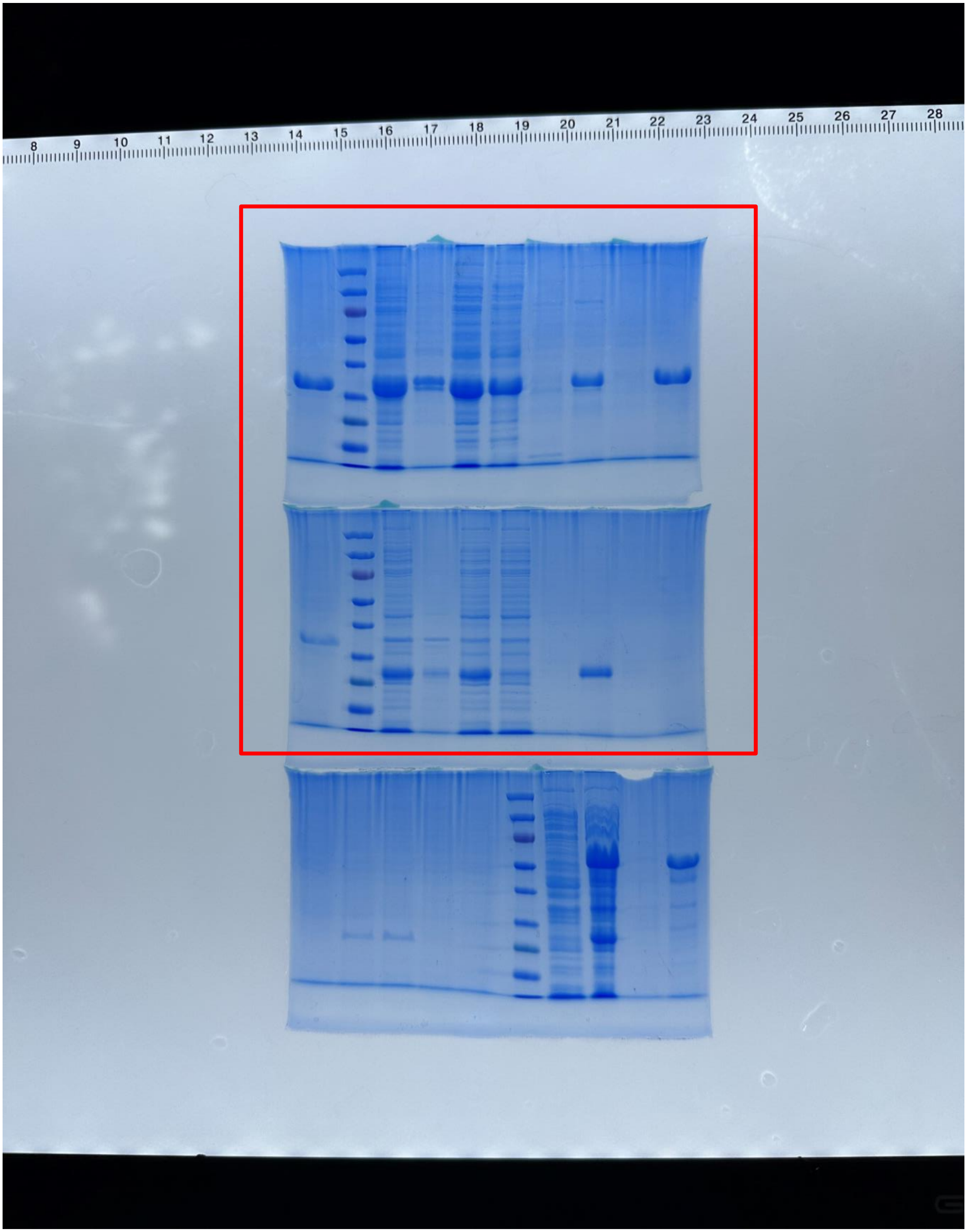


**Supplementary Figure 5.** Expression and purification of recombinant proteins SC-mi3 and ST-PCV2 Cap (Original image).

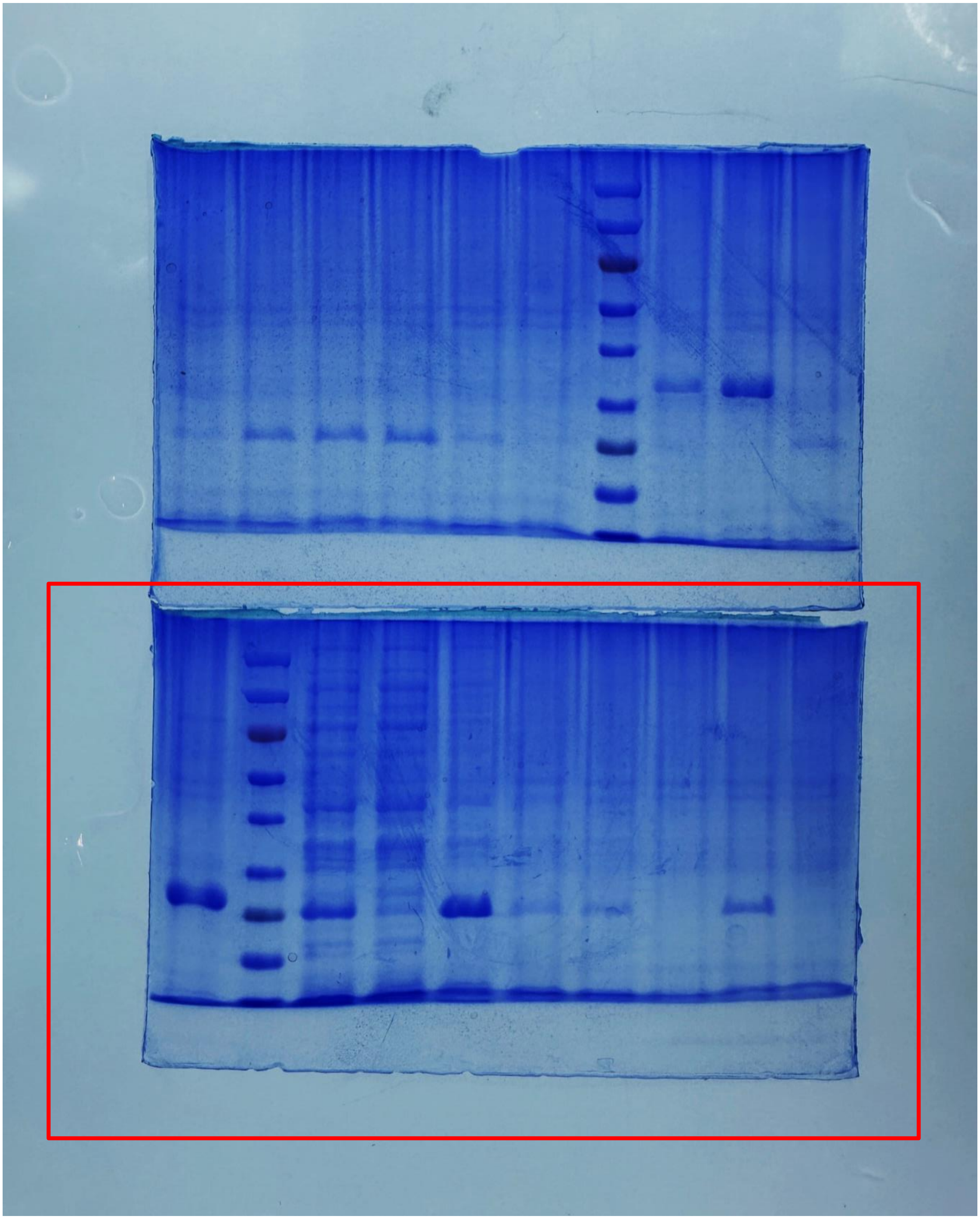

**Supplementary Figure 6.** Expression and purification of recombinant proteins ST-PCV3 Cap (Original image).

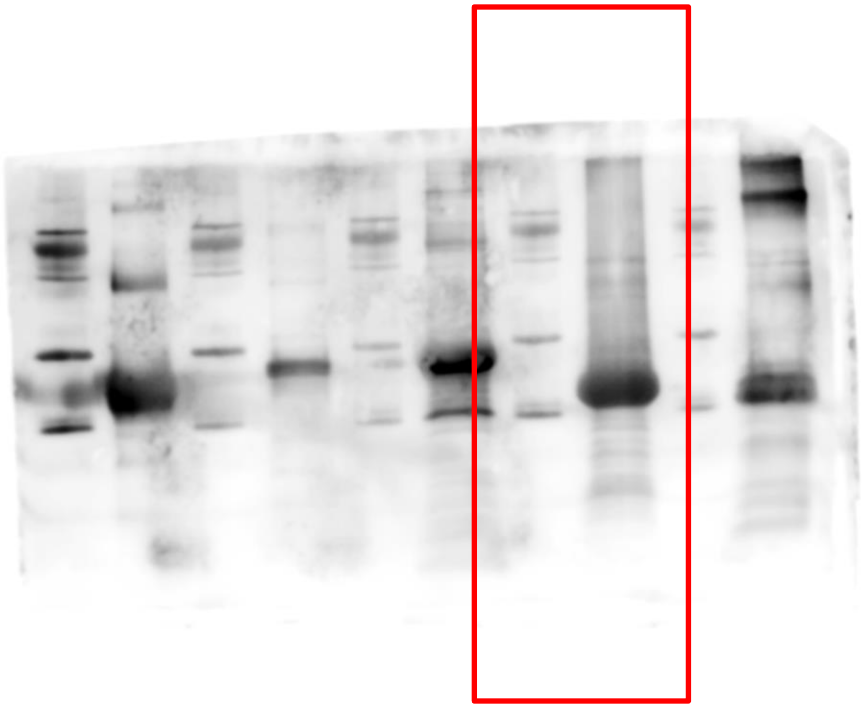

**Supplementary Figure 7.** Western blot validation of ST-PCV2 Cap (Original image).

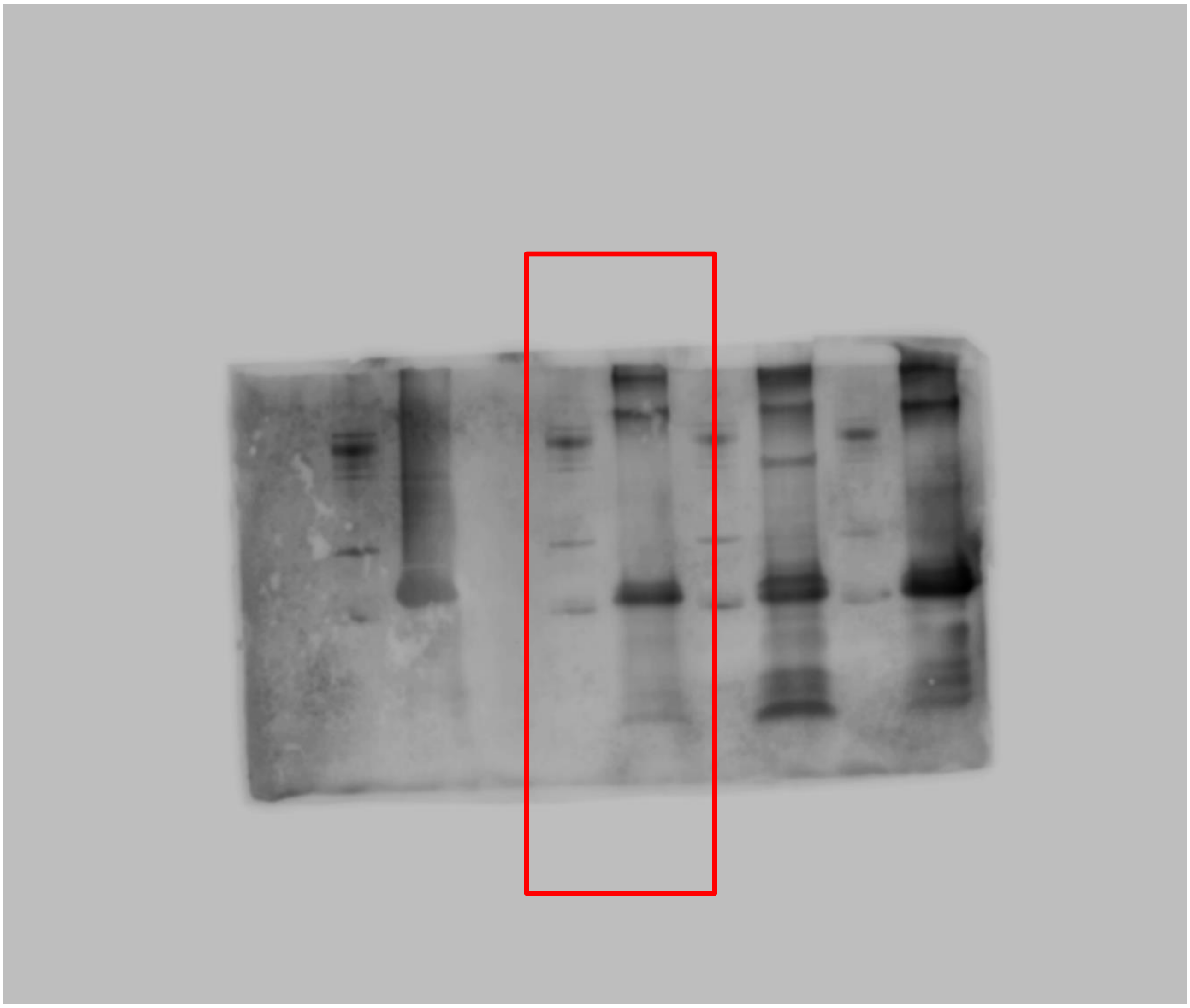

**Supplementary Figure 8.** Western blot validation of ST-PCV3 Cap (Original image).

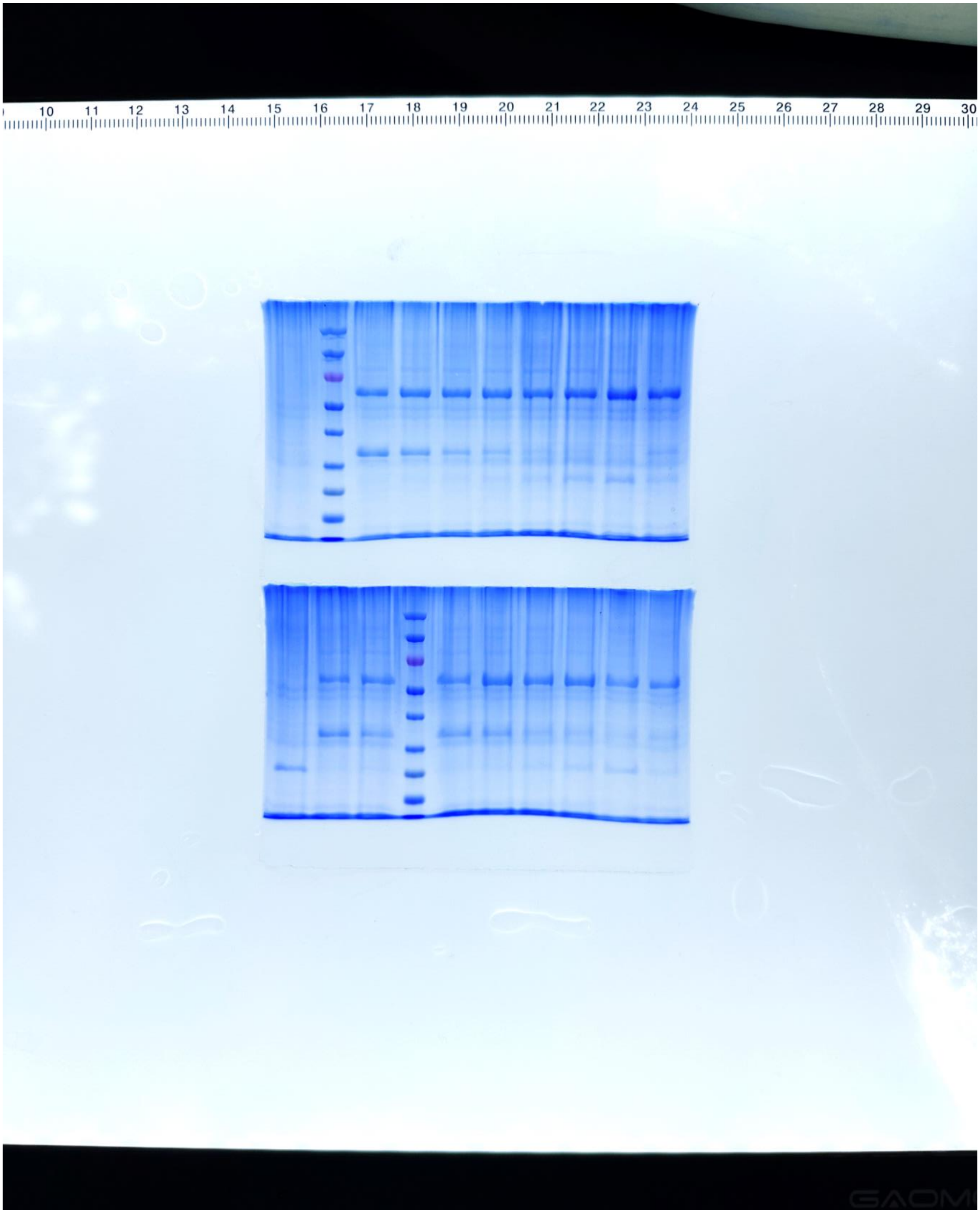


**Supplementary Figure 9.** Optimization of the molar ratio for conjugation of SC-mi3 with ST-PCV2 Cap and ST-PCV3 Cap (Original image).

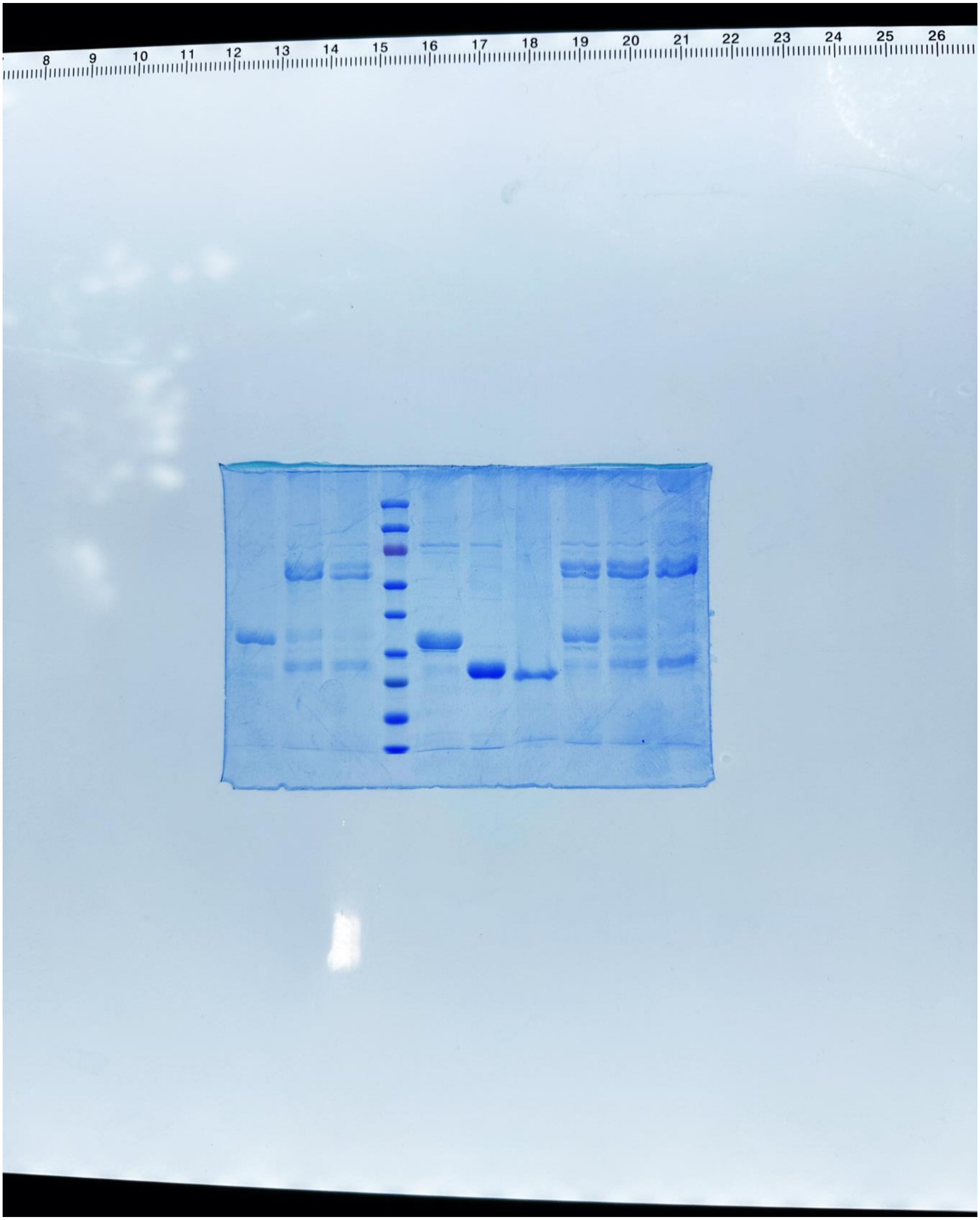


**Supplementary Figure 10.** Optimal molar ratio for the conjugation of SC-mi3, ST-PCV2 Cap, and ST-PCV3 Cap (Original image).
